# Determinants of regulatory compliance in health and social care services: a systematic review

**DOI:** 10.1093/eurpub/ckac131.286

**Published:** 2022-10-25

**Authors:** P Dunbar, F Barry, J Browne, L O'Connor

**Affiliations:** 1 Regulation Directorate, Health Information and Quality Authority, Cork, Ireland; 2 School of Public Health, University College Cork, Cork, Ireland

## Abstract

**Background:**

The delivery of high quality health and social care services is a fundamental goal for health systems worldwide. Quality is variable in services and settings. One response to variation in quality is a regulatory framework that looks to set minimum standards that are enforced by an independent public authority. This systematic review seeks to identify and describe determinants of regulatory compliance in health and social care services.

**Methods:**

Systematic searches were carried out on five electronic databases and grey literature sources. Titles and abstracts were screened by two reviewers independently. Determinants evaluated in studies were identified, extracted and allocated to constructs in the Consolidated Framework for Implementation Research (CFIR). The included studies were quality appraised by two reviewers independently. The results were synthesised narratively under each CFIR domain.

**Results:**

The search yielded 6,515 articles for screening, of which 148 were included. Most studies were quantitative designs focused on specific exposures (e.g. staffing levels, size, for-profit status). Qualitative studies were sparse, limiting investigation of the processes underlying regulatory compliance. Most of the determinants identified fit within the inner and outer setting domains of the CFIR, many with mixed findings in terms of an association with compliance. There were fewer determinants identified in the intervention characteristics, characteristics of individuals, and process domains of the CFIR.

**Conclusions:**

The literature in this field focuses on the broader concept of quality and appears to neglect the more nuanced issues surrounding the successful implementation of regulatory standards i.e. compliance. A number of gaps, particularly in terms of qualitative work focussed on the mechanism involved in implementing regulations, remain in the literature and further research in this area is needed to provide a clearer picture.

**Key messages:**

• No clear determinants of regulatory compliance were identified, suggesting it is complex and context specific.

• There are gaps in the literature around the underlying processes which contribute to the achievement of compliance that warrant research attention.

